# Decreased Joint Position Sense of the Ankle Joint Is a Risk Factor for Falls in the Elderly

**DOI:** 10.7759/cureus.51084

**Published:** 2023-12-25

**Authors:** Munekazu Kanemitsu, Tomoyuki Nakasa, Yasunari Ikuta, Nobuo Adachi

**Affiliations:** 1 Department of Orthopaedic Surgery, Akiota Hospital, Akiōta, JPN; 2 Department of Artificial Joints and Biomaterials, Graduate School of Biomedical and Health Sciences, Hiroshima University, Hiroshima, JPN; 3 Department of Orthopaedic Surgery, Graduate School of Biomedical and Health Sciences, Hiroshima University, Hiroshima, JPN

**Keywords:** risk of falls, replication error angle, elderly, falls, joint position sense of the ankle

## Abstract

Background: Falls in the elderly are common causes of morbidity, mortality, loss of independence, and poor quality of life. We hypothesized that decreased ankle position sense is one among several risk factors that might lead to falls.

Methods: A total of 54 feet from 28 patients over 65 years of age and 10 feet from five healthy volunteers were included. Measurements of ankle position sense, medical history, and fall history within a year were obtained, which were compared between the groups.

Results: The mean replication error angle of internal and external rotation was significantly higher in the elderly, and the mean replication error angle of internal rotation was significantly higher in the group with a history of falls.

Conclusion: The mean replication error angle of internal rotation and a history of fractures were significant risk factors for falls. Hence, an increase in the mean replication error angle of internal rotation may increase the risk of falls in the elderly population.

## Introduction

The increasing elderly population is a global concern. The World Health Organization reported that by 2030, one in six people worldwide will be aged 60 years or over, which indicates an increase from one billion in 2020 to 1.4 billion. By 2050, the global population over 60 years will double (2.1 billion) and the population over 80 will triple between 2020 and 2050, reaching 426 million [1]. Longer life expectancy and declining fertility rates are the main drivers of this major demographic change [2]. The expected increase in the proportion of the elderly is important from a public health perspective because aging is generally associated with a progressive decline in physical and mental health, increased risk of disability and dependence, and increased complications [3-5].

Falls are one of the most common and serious public health problems, and fall prevention is an important public health issue. Falls and injuries in the elderly are common causes of morbidity and mortality, loss of independence, and decreased quality of life [6-9]. Falls occur in 30-60% of the elderly each year, and 10-20% of these result in injury, hospitalization, and death [10]. As life expectancy increases worldwide, the health burden and costs associated with falls are increasing [11]. Several risk factors for falls have been reported, such as weakness, unsteady gait, confusion, and psychoactive medications. Muscle weakness and problems with gait and balance are the most important risk factors for falls [10]. As a cause of falls, gait problems and weakness are the second most common specific precipitating cause of falls after accidental or environmental causes [10]. Hence, assessment of gait and balance problems is important to prevent falls in older people. One study evaluated the ankle joint position sense of plantar flexion and balance in older people and concluded that decreased ankle proprioception could be a risk factor for falls in older adults [12].

To our knowledge, there have been no reports on ankle joint position sense of internal and external rotation and falls. We focused on ankle proprioception because abnormal proprioception is associated with sprains and could be a risk factor for falls. We hypothesized that decreased ankle position sense might lead to falls rather than sprains in older adults with reduced physical capacity; thus, this study aimed to analyze and investigate the relationship between ankle position sense and falls in older adults.

## Materials and methods

The study was conducted at a single institution and all eligible patients belonged to the same medical region. Patients who visited Akiota Hospital between December 2020 and March 2021 and healthy volunteers under 65 years were included in the study. Those with an inability to walk and were undergoing treatment for hip, knee, foot, and ankle fractures were excluded. All participants underwent measurements of ankle joint position sense and, in the elderly group, medical history of diabetes mellitus (DM), spinal disease, hallux valgus, fractures (proximal femoral fracture, distal radius fracture, vertebral fracture, and pelvic fracture) caused by falls, and history of falls (falls caused by external factors were excluded) within a year were obtained from interviews and medical records. In addition, the elderly group was divided into two groups according to whether they had a history of falls. These parameters were compared between the control group and the elderly group, with and without falls. This study was approved by the Ethics Committee of Akiota Hospital (approval number: R05-2). All patients were informed of the study, and their written informed consent was obtained.

Measurements of ankle joint position sense

**Figure 1:**
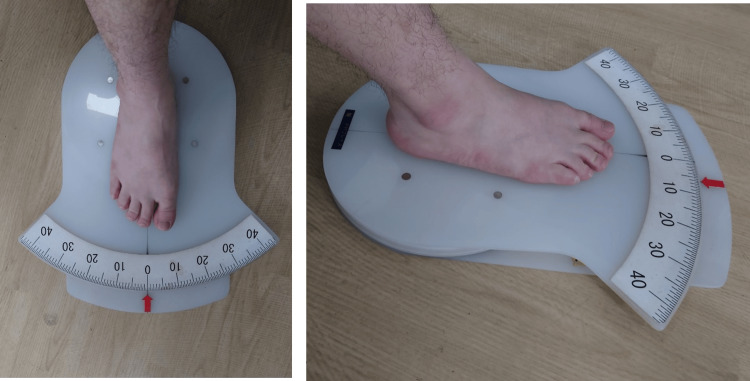
Measurement of the replication errors. Measurement of the replication errors. The foot was initially placed on a goniometer foot. The foot was passively rotated randomly from 0° internally to one of eight positions (5°, 10°, 15°, 20°, 25°, and 30° in internal rotation and 5° and 10° in external rotation).

Ankle position sensing was measured using a previously reported method [13]. The participants were seated with their knees flexed at 70° without shoes and socks. The participants placed the feet on a goniometer footplate (Nakamura Brace Co., Shimane, Japan) with the ankle at 20° plantar flexion. The goniometer footplate could rotate internally, indicating that the fulcrum of the foot motion is approximately aligned with the axis of internal and external rotation (Figure 1). The center of rotation of the goniometer footplate was just below the calcaneal tuberosity. The participants were blindfolded to eliminate visual input, improve concentration during the test, and relax their leg muscles. The foot was passively rotated randomly from 0° internally to one of eight positions (5°, 10°, 15°, 20°, 25°, and 30° in internal rotation and 5° and 10° in external rotation). The footplate was manually rotated to the index angle for approximately 1 second and held there for 5 seconds. Then, the ankle was returned to the 0° position. After that, the participants actively moved their ankles to the previous position. The difference between the index angle and replication angle was recorded as the replication error.

Statistical analysis

Statistical differences between the two groups were calculated using Welch’s t-test. Logistic regression analysis with the stepwise selection method was used to analyze the relationships between fall history and replication error, DM, spinal disease, hallux valgus, and fractures. Statistical significance was set at P<0.05 for all analyses.

## Results

Patient demographics

A total of 54 feet from 28 patients over 65 years of age (elderly group; age, 79±7.5 years; range, 66-92 years) and 10 feet from five healthy volunteers (control group; age, 35±14.7 years; range, 22-59 years) were included in this study. In the elderly group, 27 feet had a history of falls (age, 78.0±7.5 years; range, 66-92 years), while the remaining 27 did not (age, 80.8±7.4 years; range, 68-88 years).

Control Versus Elderly Group

**Figure 2:**
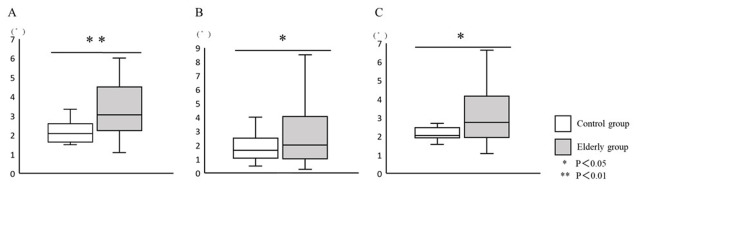
Evaluation of the ankle joint position sense between the control and elderly group. The replication error of (A) internal rotation and (B) external rotation, and (C) the general replication error.

The mean replication error angle (elderly group, 3.9±3.1°; control group, 2.1±0.4°; P=0.02), and the mean replication error angle of internal rotation (elderly group, 3.9±2.9°; control group, 2.2±0.6°; P<0.01) and external rotation (elderly group, 3.4±3.7°; control group, 1.9±1.2°; P =0.03) were significantly increased in the elderly group (Figure 2).

With Falls Versus Without Falls

**Figure 3:**
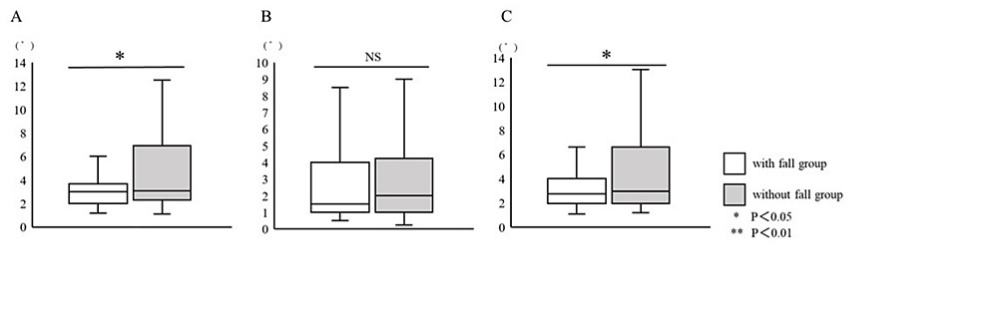
Evaluation of the ankle joint position sense between the with fall and without fall history group. The replication error of (A) internal rotation and (B) external rotation, and (C) the general replication error.

The mean replication error angle (with fall history group, 4.6±3.6°; without fall history group, 3.0±1.3°; P=0.04) and the mean replication error angle of internal rotation (with fall history group, 4.8±3.7°; without fall history group, 3.0±1.2°; P=0.03) were significantly increased in the with fall history group (Figure 3).

Risk factors for falls

Relationships between the fall history and age (with fall history: age, 78.0±7.5 years; range, 66-92 years; without fall history: age 80.8±7.4 years; range, 68-88 years), sex (with fall history: male, 10 feet; female, 17 feet; without fall history: male, two feet; female, 25 feet), spinal disease (with fall history: 13 feet; without fall history: eight feet), hallux valgus (with fall history: two feet; without fall history: five feet), DM (with fall history: six feet; without fall history: four feet), and history of fractures (with fall history: six feet; without fall history: 19 feet). Logistic regression analysis showed that the mean replication error angle of internal rotation (OR, 2.05; 95%CI, 1.325-3.175; p=0.001) and history of fractures (OR, 57.865; 95%CI, 7.035-475.988; p=0.000) were significant risk factors for falls (Table 1).

**Table 1:** Results of the logistic regression analysis. *Fractures (proximal femoral fracture, distal radius fracture, vertebral fracture, and pelvic fracture) caused by falls.

Factors				Odds Ratio	95% CI		p-value
					Lower	Upper	
History of fractures*				57.865	7.035	475.988	0.000
Replication error of internal rotation				2.051	1.325	3.175	0.001
Spinal disease				0.162	0.027	0.953	0.044

## Discussion

This study showed that the mean replication error angle of internal and external rotation was significantly higher in the elderly and that the mean replication error angle of internal rotation was significantly increased in the group with a history of falls. An increased mean replication error angle of internal rotation may increase the risk of falls in the elderly population.

Falls occur in 30-60% of the elderly, and 10-20% of these result in injury, hospitalization, and death. Understanding the risks and causes of falls is important for their prevention. Gait/balance disturbance or muscle weakness is the second most common cause of falls in the elderly after environmental factors, accounting for 3-39% of falls [10]. Normal gait involves several biomechanical factors, including free joint mobility, appropriate timing of muscle activity, appropriate intensity of muscle activity, and normal sensory inputs, including vision, proprioception, and the vestibular system [11]. Thus, it is important to assess balance in the elderly for fall prevention. The timed up-and-go or Tinetti’s gait and balance test is useful for evaluating balance [8]. However, these tests might be difficult in people over 75 years because of their declining walking ability. A large longitudinal study of persons over 75 years showed that 10% needed assistance in walking across a room, 20% were unable to climb a flight of stairs without assistance, and 40% were unable to walk half a mile [10]. The measurement technique in the current study may be useful because it is performed in a seated position, allowing for the evaluation of patients who have difficulty walking.

The results of this study indicated that a history of fracture and the mean internal rotation error angle were risk factors for falls. Other factors were not found to be associated with falls, although they were thought to be associated with decreased foot sensation and falls due to neurological damage. Falls are a cause of fractures; therefore, a strong association may have been observed. Chen et al. evaluated the relationships between lower limb joint proprioception, postural balance, and age-related differences. They concluded that decreased ankle and hip proprioception may be risk factors for falls in older adults and that ankle proprioception contributes the most to balance maintenance. In this study, we assessed ankle proprioception using ankle joint position sense. Decreased ankle joint position sense is involved in chronic ankle instability and ankle osteoarthritis. Jerosch et al. reported that the mean replication error for the unstable ankle was 2.52° [14]. Konradsen et al. evaluated 23 unilateral and functionally unstable participants and reported that the replication error value for the unstable ankle was 2.5° and for the healthy side was 2.0° [15]. Our previous study [13] showed the mean replication error for the unstable ankle was 3.4±1.0° and for the stable ankle was 2.3±0.8°.

In the current study, the reproducibility error of the control group was 2.1 ± 0.4°, which is similar to previous reports. Reproduction errors for normal subjects have been reported to range from 1° to 3°, and the present result of 2.1° is consistent with previous reports [13-15]. The replication error of the elderly group was significantly higher than that of the control group. In addition, the replication error of internal rotation was significantly higher in the group with fall history.

Joint position sensing is also involved in muscle strength. Kang et al. investigated the correlation between dorsiflexion and plantar flexion torques, plantar flexion torque and torque ratio, plantar flexion torque and ankle joint position sense, and torque ratio and ankle joint position sense [16]. In this study, joint position sense was decreased in the elderly, especially in elderly individuals with a history of falls. These results indicate that muscle strength decreases in the elderly population. It is believed that the decreased ankle joint position sense could increase the internal rotation angle during the late swing phase of walking and landing, which results in contact between the outside of the foot and the ground, leading to a fall. Low muscle strength is a common risk factor for falls, and evaluating ankle joint position sense could be a useful method for the risk evaluation of falls in the elderly. Jeno et al. reported that a fall prevention program effectively improved muscle strength, endurance, balance, and psychological aspects in elderly women with a history of falls [6]. If rehabilitation such as muscle strength training could improve joint position sense, it may be possible to reduce the risk of falls in the elderly. Further studies are needed to evaluate changes in the joint position sense of the foot during rehabilitation and other interventions.

This study has several limitations. First, the control group was small compared to the test groups. The values seem reasonable, as the replication errors of the control group were in close agreement with previous reports [12-14]. However, further examination is needed to increase the number of subjects. Second, the history of falls was based on patient interviews and may not have been accurate, although cognitive functions were preserved. Third, some patients were excluded from the study because of cognitive decline. These patients could have a higher risk of falling and even lower proprioception. Fourth, although medical records were used to confirm a history of spinal disease, it was not considered accurate because magnetic resonance imaging or other examinations were not performed in all cases. Fifth, osteoarthritis such as knee osteoarthritis and hip osteoarthritis may cause gait disturbance and be considered a fall risk, but these effects have not been considered. Further studies are needed to assess the degree of osteoarthritis. Sixth, low bone mineral density in the elderly is a risk factor for fractures and influences the incidence of fractures associated with falls. However, bone mineral density assessment has not been performed and the impact of low bone mineral density on the presence or absence of a history of fracture has not been considered. Finally, the assessment was based only on the history of falls, and analysis of other factors was not available. Many patients in this study had neuropathic conditions, such as lumbar canal stenosis and DM, and these factors may have been involved. Further studies are required to address these limitations.

## Conclusions

In the elderly, ankle position sense was decreased due to decreased muscle strength and it may be one of the risk factors for falls in given the several limitations. With falls among the elderly becoming a social problem, evaluating ankle position sense in the elderly may be a useful technique for assessing fall risk for prevention of falls. By increasing lower extremity muscle strength and proprioceptive training, in particular ankle joint position may lead to novel fall prevention in the elderly.
